# *Magnoliae flos* Downregulated Lipopolysaccharide-Induced Inflammatory Responses via NF-*κ*B/ERK-JNK MAPK/STAT3 Pathways

**DOI:** 10.1155/2022/6281892

**Published:** 2022-06-26

**Authors:** Tae-Young Gil, Bo-Ram Jin, Yun-Yeop Cha, Hyo-Jin An

**Affiliations:** ^1^Department of Pharmacology, College of Korean Medicine, Sangji University, Wonju-si, Gangwon-do 26339, Republic of Korea; ^2^Department of Rehabilitative Medicine of Korean Medicine and Neuropsychiatry, College of Korean Medicine, Sangji University, Wonju, Gangwon-do 26339, Republic of Korea

## Abstract

**Background:**

*Magnoliae flos* is the dried flower bud of *Magnolia biondii* and related plants. It has been used as a medicinal herb for the treatment of rhinitis, sinusitis, and sinus headaches. Nevertheless, the effects of *Magnoliae flos* in microbial infection or sepsis remain unclear. In this study, we investigated the anti-inflammatory effects of *Magnoliae flos* water extract (MF) in lipopolysaccharide- (LPS-) induced septic mice and LPS-stimulated RAW264.7 macrophages.

**Results:**

We found that MF reduced the mortality of LPS-challenged mice. Enzyme immunoassays and reverse transcription polymerase chain reaction analysis revealed that MF administration attenuated mRNA expression and protein production of proinflammatory mediators, including cyclooxygenase 2, inducible nitric oxide synthase, tumor necrosis factor-*α*, and interleukin-6. In parallel to these results in mice, pretreatment with MF suppressed the LPS-induced production of proinflammatory mediators in RAW264.7 macrophages. In addition, we found that MF exerted its suppressive effects by inhibiting the activation of the mitogen-activated protein kinase, nuclear factor-*κ*B, and signal transducer and activator of transcription pathways at the protein level.

**Conclusion:**

MF could be a potential therapeutic agent for regulating excessive inflammatory responses in sepsis.

## 1. Introduction

Sepsis is a severe inflammatory condition characterized by organ dysfunction in severe cases, and it is mostly caused by bacterial infection [1]. Infectious diseases in humans have been documented dating as far back as 1000 BC, but pathogenic infections still remain the leading cause of morbidity and mortality worldwide [2]. Every year, sepsis accounts for one-third of all neonatal deaths worldwide [3]. According to the World Health Organization, neonatal sepsis is a major global health concern with the highest burden in low- and middle-income countries [4].

Despite the high mortality rate of sepsis, the etiology of the disease remains unclear. Pathogens invade cells, and their toxins trigger sepsis through two processes: massive burst of proinflammatory cytokines and dysregulation of host immune responses [5–7]. The core strategy for sepsis treatment is identifying and applying antibacterial and anti-inflammatory agents to the primary lesion. Corticosteroids, such as dexamethasone (Dexa), are a class of medications used in the treatment of severe acute inflammation [8]. Although steroidal agents are effective and provide quick relief, the severe adverse effects have caused reluctance to use corticosteroids even in high-risk individuals [9].

To investigate anti-inflammatory agents, researchers have used murine models of sepsis due to their advantages, such as ease of experimentation, availability of genetically engineered species, and the relative low cost [10]. Lipopolysaccharide- (LPS-) induced endotoxemia model is one such model that has been used for nearly 100 years in an effort to recapitulate human sepsis [11]. LPS, a major component of the outer membrane of gram-negative bacteria, activates innate immune cells, such as macrophages, to secrete proinflammatory cytokines and mediators through inflammatory pathways, such as nuclear factor-*κ*B (NF-*κ*B) and mitogen-activated protein kinase (MAPK) signaling pathways [12]. MAPK pathways include extracellular signal-regulated kinase (ERK) 1/2, c-Jun N-terminal kinase (JNK), and p38, all of which are crucial regulators of NF-*κ*B and activator protein- (AP-) 1 activation [13]. LPS-activated pathways induce macrophages to produce various proinflammatory cytokines, such as tumor necrosis factor- (TNF-) *α* and interleukin- (IL-) 6, and proinflammatory mediators, such as nitric oxide (NO) and prostaglandins (PGs) [14]. Inducible NO synthase (iNOS) and cyclooxygenase- (COX-) 2 mediate inflammatory responses by synthesizing NO and prostaglandin E2 (PGE_2_), respectively [15, 16]. Therefore, the underlying molecular mechanism behind inflammatory responses may be correlated with the inhibition of NF-*κ*B and/or MAPK signaling pathways [17]. In addition, IL-1*β* and IL-6 induce tyrosine-phosphorylation of signal transducer and activator of transcription (STAT) 3, which in return induces inflammatory responses [18, 19].

*Magnoliae flos*, a flower bud of *Magnolia biondii* and related plants, has been used as a traditional medicinal herb to treat rhinitis, sinusitis, nasal congestion, and sinus headaches [20]. Additionally, it has a wide spectrum of pharmacological effects, including antiallergic [21], antibacterial, and anti-inflammatory activities, as well as a neuroprotective effect mediated by its antioxidant activity [22]. Furthermore, it contains a variety of pharmacologically active compounds, such as magnosaline, an antirheumatic and antiangiogenic agent [23], and magnolin, an antiallergic agent as lignan [24] (Table 1). However, the anti-inflammatory activities and the underlying molecular mechanisms of *Magnoliae flos* water extract (MF) have not been studied extensively. Herein, we investigated the anti-inflammatory activity of MF using LPS-challenged septic mice and LPS-stimulated RAW264.7 macrophages.

## 2. Methods

### 2.1. Chemicals and Reagent

Dulbecco's modified Eagle's medium (DMEM), fetal bovine serum (FBS), penicillin, and streptomycin were obtained from Life Technologies Inc. (Grand Island, NY, USA). LPS (*Escherichia coli*, serotype O55:B5 (Cat. No. Sigma-Aldrich, L2880), LPS O111:B4 (Cat. No. Sigma-Aldrich, L3012)), 3-(4,5-dimethylthiazol-2-yl)-2,5-diphenyltetrazolium bromide (MTT), l-N6-(1-Iminoethyl)lysine (l-NIL), N-(2-cyclohexyloxy-4-nitrophenyl)methanesulfonamide (NS398), and Griess reagent were bought from Sigma Chemical Co. (St. Louis, MO, USA). Dimethyl sulfoxide (DMSO) was purchased from Junsei Chemical Co., Ltd. (Tokyo, Japan). Primary antibodies including COX-2 (sc-1745), NF-*κ*B p65 (sc-109), I*κ*B-*α* (sc-203), STAT3 (sc-482), PARP-1 (sc-25780), p-ERK (sc-7383), p-MEK1/2 (sc-81503), MEK1/2 (sc-81504), and *β*-actin (sc-47778) monoclonal antibodies were bought from Santa Cruz Biotechnology (Santa Cruz, CA, USA). iNOS (cst #2982), p-STAT3 (Y) (cst #9145), p-JNK (cst #9251), JNK (cst #9252), ERK (cst #9102), p-I*κ*B-*α* (cst #9246), p-SEK1/MKK4 (cst #9156), and SEK1/MKK4 (cst #9152) against antibodies were obtained from Cell Signaling Technology (Danvers, MA, USA). Horseradish peroxidase-conjugated secondary antibodies and normal goat serum were got from Jackson Immuno Research Laboratories, Inc. (West Grove, PA, USA). SYBR green master mix was obtained from Applied Biosystem (Foster, CA, USA). IL-6, TNF-*α*, and glyceraldehyde-3-phosphate dehydrogenase (GAPDH). The enzyme-linked immunosorbent assay (ELISA) kits for IL-6, TNF-*α*, and PGE_2_ were obtained from R&D Systems (Minneapolis, MN, USA).

### 2.2. Preparation of Magnoliae flos Water Extraction (MF)

The dried flower buds of *M. biondii* were obtained from HANHERB (Guri city, Gyeonggi province, Republic of Korea). The dried flower buds of the plants were processed and extracted with water at 100°C for 4 h with reflux condenser. The extract was filtered with Whatman filter paper and lyophilized. The percentage yield was 13.73% *w*/*w*. MF was diluted in saline prior to treat to the RAW264.7 cells or C57BL/6 mice.

### 2.3. Cell Culture and Sample Treatment

The RAW 264.7 macrophages cell line was purchased from Korea Cell Line Bank (KCLB, Seoul, Republic of Korea). This cell line was cultured in DMEM supplemented with 10% FBS, penicillin (100 U/mL), and 1% streptomycin (100 *μ*g/mL) in 37°C and 5% CO_2_ incubator. MF was dissolved in distilled water, and the cells were treated with 50, 100, or 200 *μ*g/mL MF. The cells (1 × 10^5^ cells/mL) were stimulated with 1 *μ*g/mL of LPS for the indicated time prior to treatment with MF for 1 h.

### 2.4. Experimental Animals and Sample Treatment

Male C57BL/6 mice (6 weeks old) were obtained from Daehan Biolink Co. (Daejeon, Republic of Korea). All animals were housed in accordance with the guidelines for the care and use of laboratory animals. The guidelines were adopted and promulgated by Sangji University according to the requirements demonstrated by the National Institutes of Health. All the experimental protocols were approved based on the Institutional Animal Care and Use Committee (IACUC) of Sangji University before the beginning of the study (IACUC Animal approval protocol No.2020-10). The mice were housed in a cage and fed standard laboratory chow in the animal room with 12 h dark/light cycles and constant condition (20 ± 5°C temperature, 40–60% humidity) for a week. The mice randomly were assigned to one of five groups (*n* = 6 per group). The C57BL/6 mice were intraperitoneal injected with PBS or LPS (30 mg/kg dissolved in PBS). MF (50 and 100 mg/kg) was injected intraperitoneally 1 h before LPS injection. Survival was monitored for 108 h after LPS administration. Four hours after LPS injection, peripheral blood and liver samples were obtained from each mouse.

### 2.5. Production Assay

We evaluated the production of NO, PGE2, and pro-inflammatory cytokines following the previous study [28].

### 2.6. Quantitative Real-Time PCR Analysis (qRT-PCR)

RAW 264.7 macrophages (1 × 10^5^ cells/mL) were homogenized, and total RNA was isolated using an easy-BLUE™ total RNA extraction kit (iNtRON Biotechnology Inc., Gyeonggi-do, South Korea). cDNA was obtained using isolated total RNA (1 *μ*g), d(T)16 primer, and avian myeloblastosis virus reverse transcriptase (AMV-RT). Relative gene expression was quantified with real-time PCR (Real-Time PCR System 7500, Applied Biosystems, CA, USA) with SYBR green PCR mast mix (Applied Biosystems, CA, USA). The gene Ct values of IL-6 and TNF-*α* were normalized with the gene express 2.0 program (Applied Biosystems, CA, USA) to the Ct values of GAPDH.Values are presented as mean ± SD of three independent experiments. Oligonucleotide primers were got from Bioneer (Daejeon, Republic of Korea) (Table 2).

### 2.7. Western Blot Analysis

Protein samples were isolated from RAW264.7 macrophages using specific reagents (NE-PER Nuclear and Cytoplasmic Extraction Reagents provided Thermo; Pro-prepTM obtained from Intron biotechnology Inc.) and then separated using electrophoresis and evaluated following the previous analysis [28].

### 2.8. Statistical Analysis

Results are expressed as the mean ± SD of triplicate experiments. Statistically significant differences were determined using ANOVA and Dunnett's *post hoc* test, and *pvalues* > 0.05 indicated statistical significance.

## 3. Results

### 3.1. MF Suppressed Proinflammatory Responses in LPS-Challenged Mice

**Figure 1:**
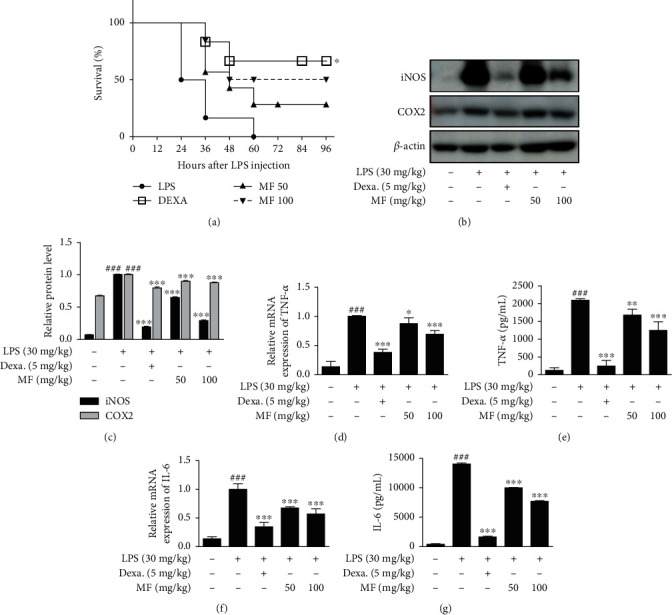
Effect of MF on proinflammatory enzymes and cytokine production in LPS-challenged mice. Mice were intraperitoneally injected with either Dexa, MF, or PBS (as a vehicle) for 1 h before intraperitoneal LPS injection (30 mg/kg) (6 mice per group). (a) Survival rates of these mice were determined 96 h postinjection. (b and c) Liver tissue samples were obtained 4 h post-intraperitoneal LPS injection. Expression of COX-2 and iNOS was determined by western blot with specific antibodies. Densitometric analysis was performed using ImageJ software. *β*-Actin was used as an internal control. (d and f) Total RNA was isolated from the liver tissue samples, and then, mRNA expression of TNF-*α* (d) and IL-6 (f) was determined with qRT-PCR. (e and g) Peripheral blood samples were obtained from each mouse. Serum levels of TNF-*α* (e) and IL-6 (g) were determined using EIA kits. The data are presented as the mean ± SD. ^###^*p* < 0.001 vs. the control group; ^∗^*p* < 0.05, ^∗∗^*p* < 0.01, and ^∗∗∗^*p* < 0.001 vs. the LPS-only-challenged group.

As one of mimicking pathology of sepsis models, LPS-made mice undergo severe inflammatory responses [5, 29]. Mice intraperitoneally injected with LPS (30 mg/kg) had 100% mortality 60 h postinjection (Figure 1(a)). Of the 6 mice in each group, 60 h postinjection, only 4 mice from Dexa group (5 mg/kg), 1 mouse from MF 50 group (50 mg/kg), and 3 mice from MF 100 group (100 mg/kg) survived, corresponding to 33.3%, 83.33%, and 50.0% mortality rates, respectively. We also observed the condition and survival of mice 96 h postinjection. Pretreatment with Dexa considerably increased the survival in mice, by more than 50%, 96 h postinjection. Since sepsis is a systemic inflammatory response induced by LPS, we investigated the effect of LPS injection on proinflammatory markers at the mRNA and protein level. As shown in Figure 1(b), the increase in iNOS and COX2 by LPS was suppressed after pretreatment with MF or Dexa the positive control. We also evaluated the levels of secreted protein and mRNA expression of proinflammatory cytokines, including TNF-*α* and IL-6 with enzyme immunoassay (EIA) and quantitative reverse transcription polymerase chain reaction (qRT-PCR), respectively (Figures 1(d)–1(g)). LPS induced an increase in mRNA expression and secreted protein level of cytokines, and this was downregulated by pretreatment with MF.

### 3.2. MF Inhibited the Activation of Proinflammatory Signals in LPS-Challenged Mice

**Figure 2:**
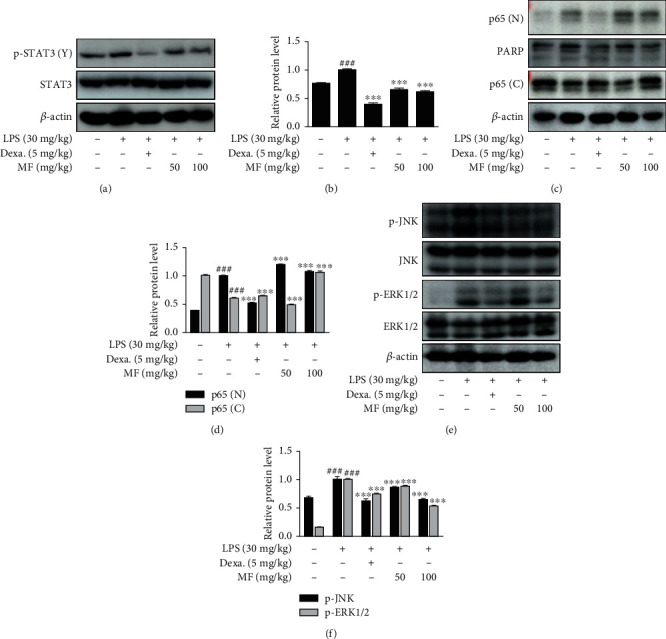
Effect of MF on LPS-induced activation of STAT3, NF-*κ*B p65, and MAPKs in LPS-challenged mice. Mice were intraperitoneally injected with either different concentrations of MF (50 and 100 mg/kg), Dexa (5 mg/kg), or vehicle (PBS), 1 h before intraperitoneal LPS injection (30 mg/kg). Liver tissue samples were obtained 4 h posttreatment with LPS, to extract protein. (a) Phosphorylated-STAT3, (c) nuclear and cytosolic NF-*κ*B p65, and (e) phosphorylation of JNK and ERK1/2 were analyzed by western blot. (b, d, and f) Densitometric analysis was performed using ImageJ software. The data are presented as mean ± SD. ^###^*p* < 0.001 vs. the control group; ^∗∗∗^*p* < 0.001 vs. the LPS-only-challenged group.

Having shown that systemic inflammatory responses induced by LPS were suppressed by pretreatment with MF (Figure 1), we further investigated the proinflammatory pathways involved in the effects of MF in liver tissue. Acute sepsis was induced in mice 4 h posttreatment with LPS (30 mg/kg). We extracted protein from mice liver tissue samples and analyzed the expression of proteins involved in proinflammatory signaling pathways using western blot. Phosphorylation of STAT3 was increased by LPS and was downregulated by pretreatment with MF (50 mg/kg and 100 mg/kg). Inhibition of STAT3 activation was also noted in the positive control, the Dexa-treated group (Figures 2(a) and 2(b)). The tendency of Dexa was presented in other results in p65 translocation and proinflammatory pathways, ERK1/2 and JNK (Figures 2(c)–2(f)). We extracted proteins using specific kits and found that LPS-induced considerable nuclear translocation of NF-*κ*B p65 showing relatively decreased cytosolic p65 content. However, MF did not inhibit on the nuclear translocation of p65 in the nucleus and cytosol fractions (Figures 2(c) and 2(d)). We also evaluated the effects of MF on the activation of MAPKs. From the 3 members of the MAPK family, MF downregulated LPS-induced phosphorylation of JNK and ERK1/2 Figures 2(e) and 2(f)) but not that of p38 (data not shown).

### 3.3. MF Inhibited Proinflammatory Mediators in LPS-Stimulated RAW264.7 Macrophages

**Figure 3:**
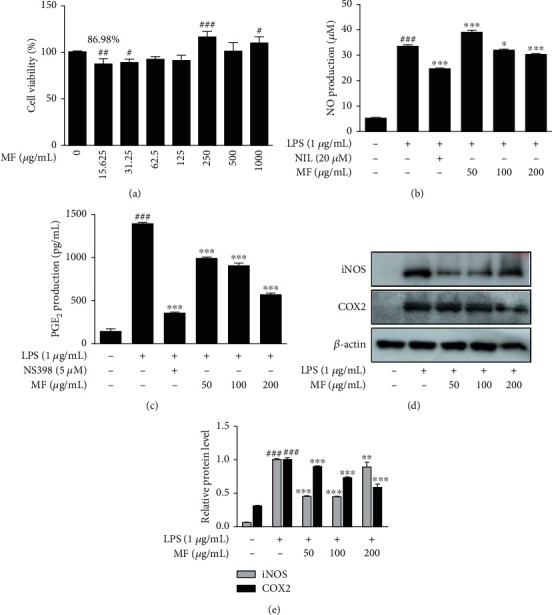
Effect of MF on proinflammatory mediators in LPS-stimulated RAW264.7 macrophages. (a) The cells were treated with various concentrations of MF for 24 h, and their viability was determined by MTT assay. (b and c) The cells were treated with 50, 100, or 200 *μ*g/mL of MF for 1 h prior to the addition of LPS (1 *μ*g/mL) and were incubated for 24 h. NO and PGE_2_ levels were determined using Griess reagent and EIA kit, respectively. NIL (20 *μ*M) and NS398 (5 *μ*M) were used as positive controls for NO and PGE_2_ inhibition, respectively. (d and e) The protein levels of iNOS and COX2 were determined by western blot with specific antibodies. Densitometric analysis was performed using ImageJ software. The data are presented as the mean ± SD. ^##^*p* < 0.01 and ^###^*p* < 0.001 vs. the control group; ^∗^*p* < 0.05, ^∗∗^*p* < 0.01, and ^∗∗∗^*p* < 0.001 vs. the LPS-treated group.

To confirm the anti-inflammatory effects of MF *in vitro*, we evaluated the effect of MF on LPS-stimulated RAW264.7 macrophages. We determined the noncytotoxic concentrations of MF, 50, 100, and 200 *μ*g/mL, using 3-[4,5-dimethylthiazol-2-yl]-2,5 diphenyl tetrazolium bromide (MTT) assay (Figure 3(a)). As another experiment for replicability, MTT assay was applied again in the range of 50, 100, 200, 400, and 800 *μ*g/mL followed by increasing result 200-800 *μ*g/mL of MF (data not shown). With the results, we determined the noncytotoxic concentrations of MF. Representative proinflammatory mediators, NO and PGE_2_, both increased following LPS treatment (1 *μ*g/mL) and were downregulated by treatment with their inhibitors, NIL and NS398, respectively (Figures 3(b) and 3(c)). The expression of both proinflammatory mediators was inhibited by pretreatment with 100 and 200 *μ*g/mL MF. We then examined the effect of MF on the enzymes which produce these proinflammatory mediators, i.e., NO and PGE_2_. As shown in Figures 3(d) and 3(e), the protein expression of both iNOS and COX2 increased following LPS treatment and was suppressed by pretreatment of macrophages with 50 and 100 *μ*g/mL MF.

### 3.4. MF Repressed mRNA Expression and Secreted Protein Levels of Proinflammatory Cytokines in LPS-Stimulated RAW264.7 Macrophages

**Figure 4:**
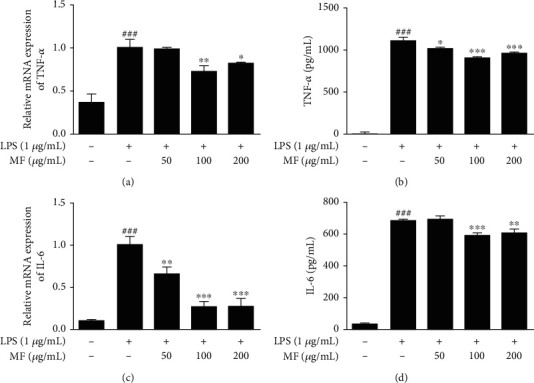
Effect of MF on proinflammatory cytokines in LPS-stimulated RAW264.7 macrophages. The cells were pretreated with MF for 1 h and were then stimulated with LPS (1 *μ*g/mL) for 6 h and 18 h to evaluate mRNA expression (a and c) and protein secretion (b and d). The data are presented as mean ± SD. ^###^*p* < 0.001 vs. the control group; ^∗^*p* < 0.05, ^∗∗^*p* < 0.01, and ^∗∗∗^*p* < 0.001 vs. the LPS-treated group.

We examined the effects of MF on the proinflammatory cytokines, such as TNF-*α* and IL-6 with qRT-PCR and EIA kits. LPS stimulation considerably increased the production of proinflammatory cytokines in RAW264.7 macrophages. However, pretreatment with MF, at 100 and 200 *μ*g/mL, downregulated mRNA expression and protein secretion of the proinflammatory cytokines, TNF-*α* (Figures 4(a) and 4(b)) and IL-6 (Figures 4(c) and 4(d)), respectively.

### 3.5. MF Inhibited Phosphorylation of STAT3 and Regulated Activation of NF-*κ*B/I*κ*B-*α* Signaling Pathways in LPS-Stimulated RAW264.7 Macrophages

**Figure 5:**
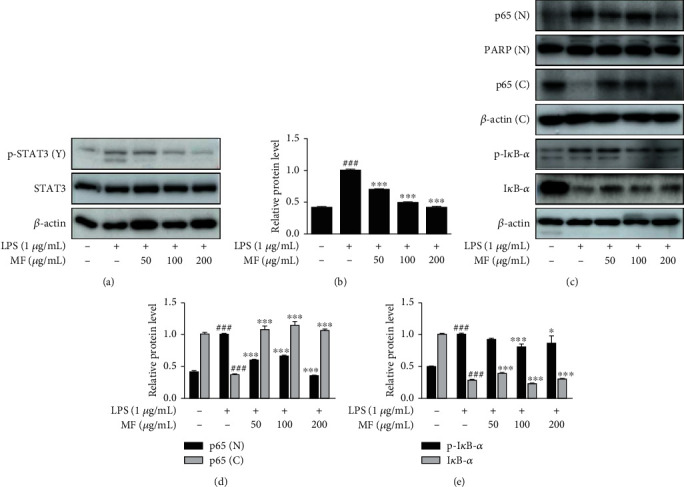
Effect of MF on phosphorylation of STAT3 and activation of NF-*κ*B/I*κ*B-*α* in LPS-stimulated RAW264.7 macrophages. The cells were pretreated with MF for 1 h prior to the addition of LPS (1 *μ*g/mL) for either 2 h (a and b) or 15–30 min (c–e). (a and b) Total proteins extracted from the cells were evaluated with specific antibodies against STAT3 and pSTAT3 by western blot. (c–e) Nuclear and cytosolic extracts were isolated, and the level of p65 in each fraction, i.e. p65 (N) and p65 (C), and LPS-induced degradation of I*κ*B-*α* was examined by western blot. The data are presented as mean ± SD. ^###^*p* < 0.001 vs. the control group; ^∗^*p* < 0.05 and ^∗∗∗^*p* < 0.001 vs. the LPS-treated group.

Given the results from LPS-challenged mice (Figures 1 and 2), we investigated 3 proinflammatory signaling pathways involved in the severe inflammatory responses. As shown in Figures 5(a) and 5(b), pretreatment with MF inhibited the phosphorylation of STAT3 induced by LPS. We also examined the effect of MF on the nuclear translocation of NF-*κ*B p65. LPS stimulation showed notable nuclear translocation of p65 from cytosol to nucleus, and this was inhibited by pretreatment with MF though the tendency was not presented in dose-dependent manner (Figures 5(c) and 5(d)). We also found that MF downregulated the phosphorylation of I*κ*B-*α* stimulated by LPS; however, the effect of MF on the activation of I*κ*B-*α* or restoration of degraded I*κ*B-*α* was not dose dependent (Figures 5(c) and 5(e)).

### 3.6. MF Reduced Phosphorylation of MEK1/2-ERK1/2 and SEK1/MKK4-JNK Pathways in LPS-Stimulated RAW264.7 Macrophages

**Figure 6:**
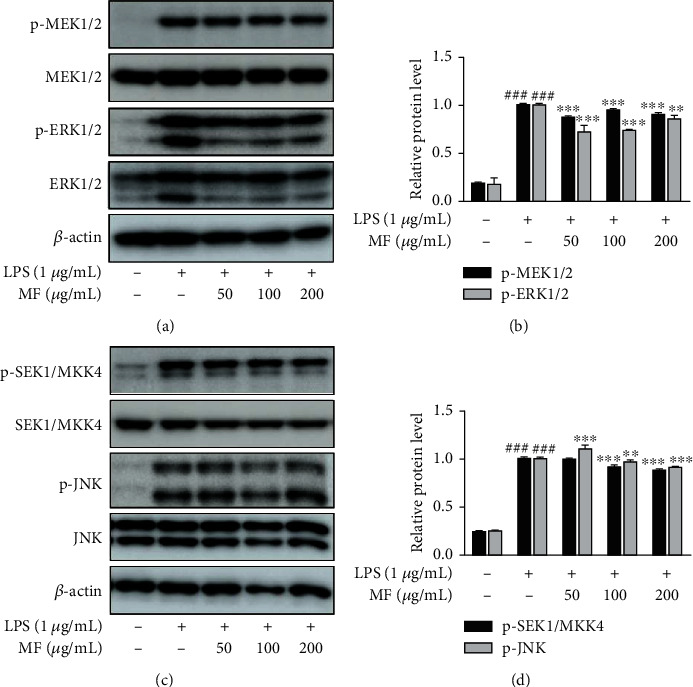
Effect of MF on phosphorylation of MEK1/2-ERK1/2 and SEK1/MKK4-JNK pathways in LPS-stimulated RAW264.7 macrophages. The cells were pretreated with MF for 1 h and then stimulated with LPS (1 *μ*g/mL) for 10–20 min for analysis of MAPKs. Phosphorylation of MEK1/2-ERK1/2 (a and b) and SEK1/MKK4-JNK (c and d) was determined by western blot. The data are presented as mean ± SD. ^###^*p* < 0.001 vs. the control group; ^∗∗^*p* < 0.01 and ^∗∗∗^*p* < 0.001 vs. the LPS-treated group.

While MF inhibited the expression of activated MAPKs in LPS-challenged mice (Figures 2(e) and 2(f)), it presented the suppressive effects of MF in the LPS-stimulated macrophages. Hence, we further investigated the effect of MF on signaling pathways, MEK1/2 and SEK1/MKK4, which are upstream to ERK1/2 and JNK, respectively. LPS-induced phosphorylation of ERK1/2 and MEK1/2 was downregulated by pretreatment with MF (Figures 6(a) and 6(b)). Moreover, LPS-induced phosphorylation of JNK and SEK1/MKK4 was also reduced by pretreatment with 100 and 200 *μ*g/mL MF (Figures 6(c) and 6(d)).

## 4. Discussion

*Magnoliae flos*, the dried flower bud of *M. biondii* and related plants, is an effective traditional remedy for rhinitis, sinusitis, and sinus headaches. The water extract of *M. biondii* has been studied previously, and compounds including magnosaline and magnolin, with antiallergic, antirheumatic, or antioxidant properties, have been identified [20–24]. In the present study, we investigated the effect of MF in LPS-challenged mice and LPS-stimulated RAW264.7 macrophages. LPS treatment in mice and murine macrophages induced excessive inflammatory responses through increased secretion of proinflammatory cytokines and mediators via activation of proinflammatory signaling pathways. Several previous reports showed anti-inflammatory activity of Magnoliae flos extracts in macrophage models. Different liquids for extract such as ethanol [30] or methanol [31] are available for keeping the anti-inflammation activity of Magnoliae flos. Also, other previous studies have presented the various suppressing inflammatory signaling pathways accompanied by antioxidant downregulating NF-*κ*B [30], ovariectomy-induced osteoporosis regulating osteoclastogenesis [32], and cancer-mediated bone destruction blocking the vicious cycle [33]. In addition, many active compounds of Magnoliae flos showed the anti-inflammatory effects such as fargesin [34], tetrahydrofurofuran-type lignans [33], or essential oil [35]. Though extraction solvent, accompanied disease or signaling pathways, or subjects on the experiments are different from this present work, all of them show the effects based on its anti-inflammatory effects.

Inflammation is necessary to maintain homeostasis in the immune system, whereas it is needed to suppress the reactions when it comes to excessive situation causing chronic and undesirable phenomenon [36]. Prolonged inflammation can lead to chronic pathological conditions, such as chronic bronchitis, which are difficult to treat, with symptoms that worsen over time [37]. Therefore, it is important to investigate and develop both prevention and treatment strategies for excessive inflammation. Sepsis, characterized by its systemic inflammatory response syndrome, is recognized as a global public health issue with high mortality and economic burden [38]. To investigate preventative and/or therapeutic agents for sepsis, we used an *in vivo* model, LPS-challenged mice [10]. Although the LPS-challenged murine model of sepsis has been used for nearly 100 years and has various advantages, there are some inherent limitations in its ability to recapitulate sepsis in humans. The bacterial infection model, bacteria and fibrin clot implantation, cecal slurry injection, and colon ascendens stent peritonitis model are some other models for studying sepsis *in vivo* [39, 40].

In the present study, we used LPS-induced murine sepsis model to investigate the anti-inflammatory properties of MF. Considering the wide spectrum of LPS dependent on doses or species, we would be able to perform other model of sepsis for further study. In mice, LPS dose of 30 mg/kg (intraperitoneal) had 100% mortality 60 h postinjection. While 67% of mice in the Dexa group and 50.0% of mice in the high-dose MF (100 mg/kg) group survived 96 h postinjection. Although it was not reflected in the survival rate (Figure 1), analysis of proteins extracted from liver tissue and serum suggests that MF can induce an anti-inflammatory effect.

As an endotoxemia model, LPS-challenged sepsis model starts with binding to Toll-like receptor- (TRL-) 4 [10]. TLR4 is an important receptor that mediates the phosphorylation of NF-*κ*B pathways through signal transduction, which involves the innate immune system. LPS-induced TLR4-mediated NF-*κ*B signaling pathway has been related to improper immune responses [41]. The pathway activates immune cells to produce proinflammatory cytokines or mediators, such as TNF-*α*, IL-6, NO, or PGE_2_. NO is distinctly produced in the inflammatory response of LPS-stimulated macrophages. As a small diffusible molecules playing a variety of physiological activities, NO is produced by activated macrophages with iNOS [42]. In addition, it seems to be produced by other proinflammatory mediators such as interferone-*γ* [43] or MF itself which resulted in significantly higher production at 50 *μ*g/mL of MF. PGE_2_ is an important mediator that increases during trauma and sepsis and enhances neutrophilic inflammation [44]. In the present study, we evaluated the induction of proinflammatory cytokines and mediators by LPS stimulation *in vivo* and *in vitro*. We found that MF suppressed the effects of LPS in the production of proinflammatory markers in LPS-challenged mice and LPS-stimulated macrophages (Figures 1, 3, and 4). These effects were confirmed with downregulation of mRNA expression of TNF-*α* and IL-6 by MF treatment (Figures 1(d), 1(f), 4(a), and 4(c)). In addition, we evaluated the effects of MF on RA264.7 macrophages depending on the noncytotoxicity concentration. In Figure 3(a), cell viability of MF treatment at 200-1000 *μ*g/mL showed proliferation effect which is assumed to be noncytotoxic condition. Based on the nontoxic and anti-inflammatory range of MF, we investigated the effects of MF on murine cell line as well as LPS-propelled sepsis animal model.

MAPK activation by LPS is a crucial signal transduction event that regulates the production of proinflammatory cytokines and mediators and the activation of NF-*κ*B [13]. MF showed inhibitory effects on the phosphorylation of MAPK or nuclear translocation of p65. While effects of MF were presented on the MAPKs, the effects of MF on nuclear translocation of p65 showed discrepancy and increased nuclear p65 level in mouse liver whereas suppressed nuclear translocation of p65 in murine cell line. We assumed that it is caused by lack of time let the subjects under the stimuli (Figure 2(d) and 5(c)) [5]. Meanwhile, the representative endotoxin, LPS, triggers excessive production of the STAT protein family, specifically STAT3, which is associated with the upregulation of LPS-binding protein, causing an amplification of inflammation in sepsis [45]. We also found that MF suppressed signaling pathways in the nucleus and the cytosol in LPS-stimulated cells and mice (Figures 2, 5, and 6). However, further work should be conducted to investigate the effect of MF on the translocation of STAT3 or LPS-stimulated inflammatory responses in other organs.

## 5. Conclusion

**Figure 7:**
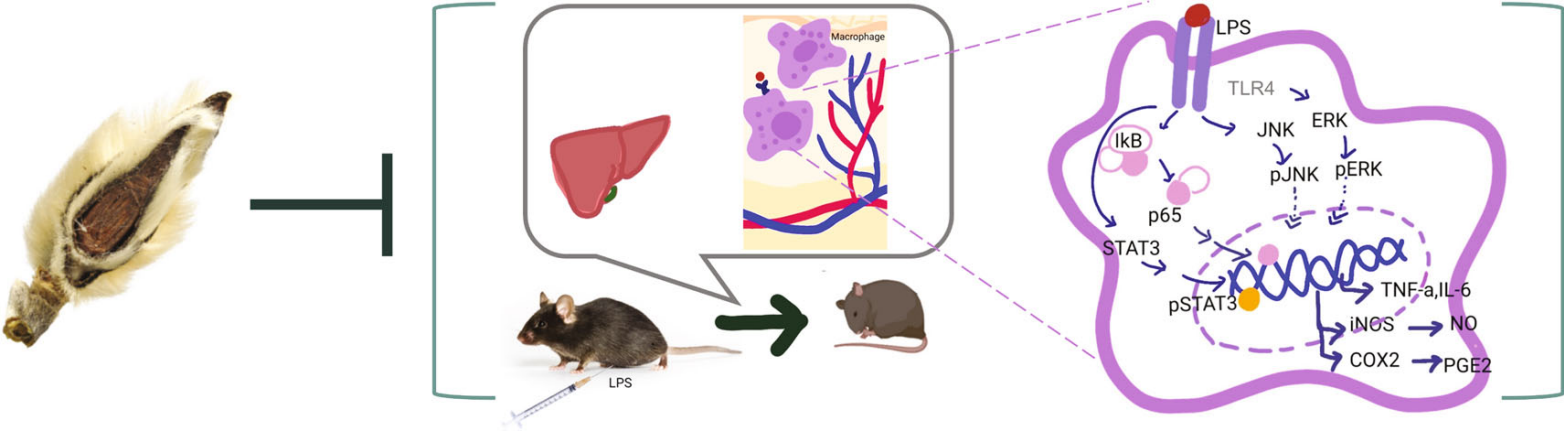
Schematic summarizing the cell signaling cascades affected by MF in LPS-induced inflammatory responses in septic model and RAW264.7 macrophages.

In conclusion, we reported that MF decreased mortality in LPS-challenged mice and suppressed the expression of proinflammatory cytokines and related proteins. MF also decreased production and expression of proinflammatory markers in LPS-stimulated macrophages. The suppressive effects of MF on the inflammatory responses seem to be regulated by the NF-*κ*B/JNK-ERK MAPK/STAT3 pathways. Therefore, we suggest the possibility of MF as an anti-inflammatory agent (Figure 7).

## Figures and Tables

**Table 1:** Active compound of *Magnolia biondii.*

Active compound	PubChem CID	References
Magnolin	169234	[24–26]
Fargesin	10926754	[27]

**Table 2:** Primers.

Gene	Primer sequences	
TNF-*α* (mouse)	Forward (5′-3′)	ATGAGCACAGAAAGCATGAT
	Reverse (5′-3′)	TACAGGCTTGTCACTCGAAT

IL-6 (mouse)	Forward (5′-3′)	TTCCATCCAGTTGCCTTCTTG
	Reverse (5′-3′)	GGAGTGGTATCCTCTGTGAGTC

GAPDH (mouse)	Forward (5′-3′)	CCCACTCTTCCACCTTCGAT
	Reverse (5′-3′)	CCACCACCCTGTTGCTGTAG

## Data Availability

The data used to support the findings of this study are available from the corresponding author upon request.
